# The implications of somatic symptom disorder on the impairment of daily life are greater in post-COVID syndrome than in asthma or COPD - results of a cross-sectional study in a rehabilitation clinic

**DOI:** 10.1038/s41598-025-96055-x

**Published:** 2025-04-05

**Authors:** Antonius Schneider, Alexander Hapfelmeier, Anna Greißel, Matthias Limbach, Gabriele Schwarzl, Franziska Ebert, Veronika Huber, Markus C. Hayden

**Affiliations:** 1 Institute of General Practice and Health Services Research, Department of Clinical Medicine, TUM School of Medicine and Health, Technical University Munich, Orleansstraße 47, 81667 Munich, Germany; 2 Institute of AI and Informatics in Medicine, TUM School of Medicine and Health, Technical University Munich, Munich, Germany; 3 Klinik Bad Reichenhall der Deutschen Rentenversicherung Bayern Süd, Bad Reichenhall, Germany

**Keywords:** SARS-CoV2-infection, post-COVID, Somatic symptom disorder, Asthma, COPD, Quality of life, Viral infection, Chronic obstructive pulmonary disease, Diseases, Health care, Medical research

## Abstract

The aim was to compare the relationship between somatic symptom disorder (SSD), anxiety, depression, clinical symptoms, and daily life impairment (DLI) in post-COVID syndrome (PCS), asthma and chronic obstructive pulmonary disease (COPD). In a cross-sectional study, 371 patients (161 PCS, 121 asthma, 89 COPD) of a pulmonary rehabilitation clinic received the questionnaires PHQ-15 (Patient Health Questionnaire-15) and SSD-12 (Somatic Symptom Disorder-12) to determine SSD, GAD-7 (Generalized Anxiety Disorder-7) to determine anxiety disorder, and PHQ-9 (Patient Health Questionnaire-9) to determine depression. Lung function was estimated using whole-body plethysmography. Predictors for DLI were assessed by regression models and ROC analyses. Association of SSD with DLI was stronger in PCS (odds ratio 13.8; 95% confidence interval 1.7-109.9) than in asthma (8.5; 2.4–30.1), and was not significant in COPD (1.9; 0.5–7.5). In asthma and COPD, strongest predictors were GAD-7 (15.0; 1.9-116.8) and PHQ-9 (8.9; 1.1–71.8), respectively. Diffusion capacity was predictive in COPD (0.947; 0.916–0.979) and asthma (0.967; 0.943–0.993), but not in PCS. To conclude, SSD appears to have greater impact on DLI in PCS than asthma or COPD patients. This should be recognized appropriately during rehabilitation. Furthermore, increased psychological comorbidity should also be considered and adequately treated in asthma and COPD if necessary.

**Supplementary Information:**

The online version contains supplementary material available at 10.1038/s41598-025-96055-x.

## Introduction

SARS-CoV-2 (severe acute respiratory syndrome coronavirus type 2) is a virus that primarily enters via the respiratory tract and can infect almost all organs via the ACE-2 receptors in a short period of time, triggering COVID-19 (coronavirus disease 2019)^1^. Even today, both diagnosis and treatment of post-infectious sequelae of COVID-19 are still very challenging. Post-COVID syndrome (PCS) is defined as the persistence or new onset of symptoms three months after the infection that leads to a significant daily life impairment (DLI)^2,3^. Long-COVID syndrome is a disease lasting four to 12 weeks after the acute infection, although the terms are often used inconsistently. The most common persistent symptoms are described by the triad of fatigue, shortness of breath, and impaired cognition^4,5^. However, it is still unclear to which specific causes these PCS symptoms can be attributed. Numerous pathological changes have already been described, particularly in the heart, lungs, and brain^6–8^, increased inflammatory biomarkers, and persistent immune responses^9,10^. However, there are no specific biomarkers for the disease. Therefore, the origin of PCS is still unclear, and the clinical picture of PCS has also been considered a medically unexplained syndrome, especially in relation to the symptoms of fatigue^11–13^. From the perspective of psychosomatic medicine, medically unexplained syndromes can also be described as a somatic symptom disorder (SSD). SSD is a comparatively new diagnosis in the Diagnostic and Statistical Manual of Mental Disorders (DSM-5) and the International Classification of Diseases (ICD-11)^14^. SSD is described by three criteria^14^. The A criterion requires one or more distressing or disabling somatic symptoms. The B criterion requires disproportionate and persistent thoughts about the severity of one’s symptoms, a high level of anxiety about one’s health or symptoms, or an excessive expenditure of energy or time on these symptoms or health concerns. The symptoms should last longer than 6 months (C criterion). The diagnostic criteria differ from the previous categories of somatization disorder, as the absence of organ pathology is no longer required as a diagnostic criterion. Therefore, SSD can be diagnosed in addition to any comorbid somatic disease, thus avoiding both mind − body dualism and equating medically unexplained with psychogenic^14^. The new criteria are beneficial in reducing stigma and better targeting appropriate support services. Regarding PCS, previous studies have shown that SSD^15,16^ and psychological factors, particularly anxiety and depression^17^, are associated with symptom persistence and DLI after SARS-CoV-2 infection.

So far, however, it is unclear to which extent SSD contributes to DLI in patients with PCS and whether there are differences in this respect compared to other diseases. Based on the premise that ultimately all chronic diseases are associated with physical and psychological distress, in line with the bio-psycho-social model, a comparison of the disease patterns of PCS with other chronic diseases of the respiratory system, namely asthma and COPD (chronic obstructive pulmonary disease), could improve our understanding of PCS, which could enable optimization of therapy. Asthma is a chronic inflammatory airway disease that is often caused by allergens and is associated with reversible airway obstruction^18^. Psychological aspects, in particular anxiety disorder, play an important role and are associated with a less favorable course of the disease^19,20^. In COPD, the airway obstruction is irreversible and the chronic inflammatory reaction is very often caused by tobacco use, occasionally also by other exogenous noxious agents^21^. Psychological comorbidity in the form of anxiety and depression is also of great importance for the course of the disease^22–24^.

The study aimed to compare the relationship of somatic symptom disorder, anxiety, depression, and clinical symptoms to DLI in PCS, asthma, and COPD, controlling for the lung function parameters forced expiratory volume in 1 s (FEV1), vital capacity (VC), and diffusion capacity for carbon monoxide (DLCO). The primary hypothesis was that SSD, depression, and anxiety have a stronger relationship to DLI in PCS than in patients with asthma or COPD. A secondary aim was to compare the quality of life between these three clinical entities to gain a better understanding of the burden of PCS.

## Methods

The study was designed as a cross-sectional study. It was conducted from June 2022 to April 2023 at the Klinik Bad Reichenhall, a pulmonary rehabilitation clinic owned by German pension fund (Deutschen Rentenversicherung Bayern-Süd; DRV Bayern-Süd). The study was registered in the German Register of Clinical Studies (DRKS-ID: DRKS00029236). It was approved by the Ethics Committee of the Medical Faculty of the Technical University of Munich (2022-199-S-SR).

Participation in the study was voluntary; neither refusal nor a later revision of the willingness to participate was associated with negative consequences for treatment and/or follow-up care. Inclusion criteria were a minimum age of 18, admission diagnosis of PCS, asthma or COPD, and sufficient German language skills. In Germany, inpatient rehabilitation is offered for patients with severe chronic diseases that could potentially lead to a reduction in work capacity. The diagnosis of PCS was accepted as a hospital admission diagnosis in the presence of persistent symptoms. The diagnoses of asthma or COPD were confirmed clinically by a pulmonologist on admission, using lung function investigation with whole-body plethysmography^18,21^. The rehabilitation program is structured in accordance with the national and international recommendation guidelines for pulmonary rehabilitation^18,21^. It comprises medical diagnostics and supervision, a graded physical exercise program, respiratory physiotherapy, patient education, and psychosocial support. The program is tailored to each patient’s individual needs. Furthermore, patients with PCS received psychological group therapy to help patients cope with their disease, and neurocognitive training sessions if symptoms of cognitive impairment persisted. All three rehabilitation programs are scheduled for 3 weeks.

Patients were informed about the study during the initial medical consultation and asked to participate. They received standardized written patient information which included general information about the study and its purpose as well as details on voluntariness, right of withdrawal, and data protection. All patients who agreed to participate completed a written informed consent form. The diagnoses were collected as part of routine admission diagnostics so that no additional effort was required from the patients. The aim was to include 200 patients with PCS,100 patients with asthma, and 100 with COPD. Given these sample sizes, even very low diagnostic accuracies – measured by the area under the receiver operating characteristic curves – of 0.58 and 0.61 would be detectable with a power of 80% at the 5% significance level under the conservative assumption of a low prevalence of 10% for the investigated outcome^25^.

### Data collection and questionnaires

Patients were given a list of the most common post-COVID symptoms^3^. Patients were asked to select each symptom that they experienced and that they subjectively attributed to the SARS-CoV-2 infection or asthma or COPD. Beyond that, patients were asked if they experienced DLI due to the SARS-CoV-2 infection or asthma or COPD. The response options were ‘no limitation’, ‘persistent sick leave’, ‘retired due to COVID-19 infection’, ‘I cannot do my everyday tasks as well as before the infection (e.g. shopping, household)’, ‘I cannot be as active in my leisure activities and hobbies as before the infection’. Multiple answers could be given. Beyond that, sociodemographic data like family status, education, and employment were inquired.

SSD is operationalized via the combined application of the Patient Health Questionnaire-15 (PHQ-15) and the Somatic Symptom Disorder-B Criteria Scale (SDD-12). The PHQ-15 assesses the presence and severity of frequent somatic symptoms (A-criterion according to DSM-5) within the last 4 weeks using 15 items. Each item refers to a symptom and is scaled as being ‘Not bothered at all’, ‘Bothered a little’ or ‘Bothered a lot’^26^. The SSD-12 is a questionnaire consisting of 12 items to assess the B-criteria of SSD according to DSM-5 (psychological symptom burden in SSD). Each item is scaled on a 5-point Likert-scale ranging from 0 to 4 (0 = ‘never’, 1 = ‘rarely’, 2 = ‘sometimes’, 3 = ‘often’, 4 = ‘very often’)^27^. When combining the PHQ-15 and SSD-12 in screening for SSD, good diagnostic accuracy was achieved by applying a cut-off of ≥ 9 points in the PHQ-15 and ≥ 23 points in the SSD-12^27^.

Depression was captured with the Patient Health Questionnaire-9 (PHQ-9). The PHQ-9 comprises nine items that are scaled on a 4-point Likert-scale ranging from 0 (‘Not at all’) to 3 (‘nearly every day’). For the analysis, the item scores are summed up^28^. The critical cut-off value is reported to be 10^29^. A potentially existing comorbid anxiety disorder was determined with the Generalized Anxiety Disorder 7 (GAD-7) questionnaire. It comprises 7 Items that are scaled and analyzed such as the ones of the PHQ-9. Scores ≥ 5, ≥ 10, and ≥ 15 represent mild, moderate, and severe symptom severity, respectively^30^.

Potential chronic fatigue or fatigue symptoms were assessed using the Fatigue Assessment Scale (FAS). The questionnaire consists of 10 items scaled on a 5-point Likert scale (1 =’never’, 2 = ‘sometimes’, 3 = ‘regularly’, 4 = ‘often’, 5 = ‘always’). A sum score of ≥ 22 to 34 points corresponds to mild to moderate fatigue. Scores ≥ 35 imply severe fatigue^31^.

The European Quality of Life 5 Dimensions 5 Level Version (EQ-5D-5 L) questionnaire measures the 5 dimensions of mobility, self-care, usual activities, pain/discomfort, and anxiety/depression. The range extends from 0 (‘very poor’) to 1 (‘best possible state of health’). Another component of this questionnaire is the visual analog scale (EQ-VAS), scaled from 0 (minimum) to 100 (maximum quality of life). In COPD patients, the relevant clinical difference (‘minimally important difference’) was 0.051 for the EQ-5D-5 L and 6.9 for the EQ-VAS^32^.

Pulmonary function measurement was carried out as part of the routine examination using whole-body plethysmography. We assessed the following lung function parameters: forced expiratory volume in 1 s (FEV1), vital capacity (VC), and the measurement of the diffusion capacity of carbon monoxide (DLCO)^33^. In addition, pre-existing diagnoses, and the basic data age, gender, and BMI were extracted from the patient file.

### Data analysis

The distribution of baseline data, psychometric scales, and lung function parameters is presented using descriptive statistics. Group differences were tested for statistical significance using Mann-Whitney-U tests and chi-square tests. To determine the predictive value of the questionnaires and symptoms for DLI, univariable binary logistic regression models were calculated to estimate the odds ratios with 95% confidence intervals. DLI was dichotomized into ‘at least one impairment present’ versus ‘no impairment present’. DLI served as the dependent variable. The questionnaire scales were dichotomized using the above cut-off values and served as independent variables in separate models. Beyond that, the most common PCS symptoms ´fatigue´, ´shortness of breath´, and ´impaired cognition´^4,5^ were used as independent variables. For this purpose, the symptoms ´concentration disorder´ and ´memory disorder´ were combined to form the variable ´impaired cognition´. An interaction effect between the predictor and a group indicator was included in each regression model to test for group differences. Receiver operating characteristics curves (ROC) were used to quantify the diagnostic performance of predictors for the prediction of everyday life impairment on their original scale, i.e. without dichotomization. In addition, the combined performance of multiple predictors was assessed using the predictions of the respective multiple binary regression models in this ROC analysis. The resulting areas under the curves (AUC) were compared between the groups using the DeLong test^34^. AUC values ≥ 0.9 indicate ‘outstanding’, ≥ 0.8 ‘excellent’, and ≥ 0.7 ‘acceptable’ discrimination^35^. All data available for each analysis was used in case of missing values. Statistical analysis was performed using R 4.1.3 (The R Foundation for Statistical Computing, Vienna, Austria) and SPSS 28.0 (IBM Corp., Armonk, NY).

## Results

During the study period, a total of 438 patients with PCS, 488 patients with asthma, and 396 patients with COPD were referred to the clinic, of which 161 (36.8%) PCS patients, 121 (24.8%) patients with asthma, and 89 (22.5%) patients with COPD were recruited. There were essentially no relevant differences in the distribution of gender and age between responders and non-responders (*p* > 0.05 for gender for all three groups) (not in table). The only significant difference between responders (mean 51.79 years; standard deviation 10.65 years) and non-responders (mean 54.99; standard deviation 10.69) concerned the age distribution of patients with asthma (*p* = 0.031).

The group comparison showed that the PCS patients were younger and more often female than the other two patient groups, more often obese, and had more pre-existing mental illnesses (Table 1). Patients with COPD were more likely to have a lower level of education, had a higher incidence of diabetes mellitus, and more cardiovascular and malignant diseases. DLI was higher in PCS patients than in COPD patients and higher in the latter than in asthma patients. The incidence of fatigue and shortness of breath was high in all patient groups (Table 2). Concentration and memory disorders were particularly common in PCS.

**Table 1:** Patient characteristics.

Total *N* (%)	PCS	Asthma	COPD
371 (100)	*n* = 161 (43.4)	*n* = 121 (32.6)	*n* = 89 (24.0)
Sex female (%)	71 (44.1)	38 (31.4)	35 (39.3)
Age in years (m ± sd)	49.8 ± 9.9	51.8 ± 10.7	59.1 ± 7.1
BMI (m ± sd)	29.9 ± 5.9	28.9 ± 5.8	27.8 ± 6.9
Family status*			
Single (%)	44 (27.3)	30 (24.8)	31 (34.8)
Relationship (%)	116 (72.0)	91 (75.2)	57 (64.0)
Education*			
< 10 years (%)	80 (49.7)	59 (48.8)	68 (76.4)
Middle school (%)	48 (29.8)	36 (29.8)	14 (15.7)
Higher school (%)	32 (19.9)	24 (19.8)	6 (6.7)
Active employment (%)*	128 (79.5)	115 (95.0)	56 (62.9)
Smoking status*			
Never (%)	76 (47.2)	59 (48.8)	3 (3.4)
Former smoker (%)	71 (44.1)	51 (42.1)	46 (51.7)
Active smoker (%)	12 (7.5)	8 (6.6)	36 (40.4)
Comorbidity**			
Obesity (BMI > 30) (%)	71 (44.1)	41 (33.9)	25 (28.1)
Diabetes mellitus (%)	11 (6.8)	10 (8.3)	13 (14.6)
Cardiovascular comorbidity (%)	51 (31.7)	26 (21.5)	38 (42.7)
Respiratory comorbidity (%)	54 (33.5)	121 (100)	89 (100)
Malignoma (%)	8 (5.0)	6 (5.0)	11 (12.4)
Venous thrombosis (%)	5 (3.1)	2 (1.7)	2 (2.2)
Preexisting mental comorbidity (%)	38 (23.6)	14 (11.6)	16 (18.0)
Status post COVID-19	161 (100)	90 (74.4)	47 (52.8)
Status post second COVID-19	18 (11.2)	6 (5.0)	4 (4.5)
COVID-19 severity*			
Asymptomatic (%)	9 (5.6)		
Ambulatory treatment (%)	122 (75.8)		
Inpatient treatment (%)	18 (11.2)		
Intensive care (%)	11 (6.8)		
Treatment before rehabilitation**			
None (%)	4 (2.5)	11 (9.1)	1 (1.1)
General practitioner (%)	139 (86.3)	80 (66.1)	73 (82.0)
Specialist (%)	91 (56.5)	85 (70.2)	70 (78.7)
Naturopath (%)	24 (14.9)	7 (5.8)	2 (2.2)
Long COVID outpatient clinic (%)	15 (9.3)	-	-
Daily life impairment**			
I can no longer do my everyday tasks well. (%)	117 (72.7)	39 (32.2)	48 (53.9)
I can no longer actively organize my leisure activities. (%)	123 (76.4)	54 (44.6)	45 (50.6)
I am retired. (%)	2 (1.2)	0	7 (7.9)
I am on sick leave. (%)	84 (52.2)	13 (10.7)	24 (27.0)
At least one daily life impairment (%)	147 (91.3)	73 (60.3)	73 (82.0)

*m*  mean,* sd* standard deviation.

* Some data missing; percentages are calculated on the basis of the respective total number.

** Multiple responses possible.

**Table 2:** Subjective symptoms of patients.

Total *N* (%)	PCS	Asthma	COPD
371 (100)	*n* = 161 (43.4)	*n* = 121 (32.6)	*n* = 89 (24.0)
Fatigue	153 (95.0)	77 (63.6)	57 (64.0)
Concentration disorder	138 (85.7)	48 (39.7)	37 (41.6)
Memory disorder	133 (82.6)	36 (29.8)	27 (30.3)
Shortness of breath	126 (78.3)	78 (64.5)	70 (78.7)
Sleep disorder	83 (51.6)	58 (47.9)	45 (50.6)
Muscular pain	80 (49.7)	31 (25.6)	24 (27.0)
Vertigo	80 (49.7)	34 (28.1)	23 (25.8)
Headache	77 (47.8)	36 (29.8)	18 (20.2)
Chest pain	73 (45.3)	36 (29.8)	12 (13.5)
Cough	71 (44.1)	65 (53.7)	56 (62.9)
Depression	64 (39.8)	18 (14.9)	28 (31.5)
Anxiety	60 (37.3)	19 (15.7)	22 (24.7)
Palpitations	57 (35.4)	26 (21.5)	18 (20.2)
Distorted sense of smell	47 (29.2)	20 (16.5)	13 (14.6)
Distorted sense of taste	45 (28.0)	19 (15.7)	12 (13.5)
Tinnitus	41 (25.5)	18 (14.9)	14 (15.7)
Weight loss	19 (11.8)	8 (6.6)	20 (22.5)
Skin rashes	18 (11.2)	12 (9.9)	8 (9.0)
Loss of appetite	9 (5.6)	9 (7.4)	9 (10.1)

PCS patients had a significantly lower quality of life compared to patients with asthma and COPD (Table 3). In addition, the sum scores of the PHQ-15, PHQ-9, and FAS scales were highest in PCS patients. In the SSD-12 scale, the scores were comparable between patients with PCS and patients with COPD, but lower in patients with asthma. Most lung function values were better in patients with PCS than with asthma, which in turn were better than those with COPD.

**Table 3:** Patient questionnaires and lung function parameters in the patient groups.

	PCS m (sd)	Asthma m (sd)	COPD m (sd)	*p*-value PCS vs. Asthma	*p*-value PCS vs. COPD
Questionnaire					
EQ-5D-5 L (index value)	0.64 (0.27)	0.83 (0.17)	0.72 (0.28)	< 0.001	0.002
EQ-5D-5 L VAS	53.14 (19.01)	65.11 (14.25)	53.47 (19.46)	< 0.001	0.868
PHQ-15	13.35 (5.59)	9.63 (4.84)	10.19 (5.74)	< 0.001	< 0.001
SSD-12	24.11 (0.86)	17.17 (0.51)	22.76 (10.68)	< 0.001	0.338
PHQ-9	11.04 (5.34)	6.65 (4.88)	8.08 (5.56)	< 0.001	< 0.001
GAD-7	8.87 (13.50)	6.22 (9.54)	6.27 (5.08)	0.006	0.139
FAS	32.31 (8.94)	24.19 (8.20)	25.90 (8.29)	< 0.001	< 0.001
Spirometry					
FEV1 (l)	3.12 (0.96)	2.93 (0.92)	1.55 (0.71)	0.094	< 0.001
FEV1%pred.	87.22 (21.28)	80.80 (19.96)	47.84 (19.23)	0.002	< 0.001
VC (l)	4.38 (7.63)	4.53 (8.03)	2.72 (0.86)	0.795	< 0.001
VC %pred.	87.34 (18.57)	85.26 (17.02)	66.96 (16.31)	0.315	< 0.001
FEV1/VC	98.42 (14.51)	93.59 (13.44)	69.81 (17.59)	< 0.001	< 0.001
Diffusion capacity for carbon monoxide					
DLCO (mmol/min/kPa)	8.42 (18.50)	10.15 (10.14)	5.40 (2.29)	0.044	< 0.001
DLCO %pred.	94.86 (20.65)	99.86 (19.37)	63.36 (22.88)	0.084	< 0.001

*m * mean,* sd * standard deviation,* %pred * percentage of the predicted value,* EQ-5D-5 L* European quality of life 5 dimensions 5 level version,* FAS*  fatigue assessment scale,* GAD-7*  generalized anxiety disorder 7,* PCS*  Post-COVID syndrome,* PHQ-9*  Patient health questionnaire 9,* PHQ-15*  patient health questionnaire 15 ,*SSD-12*  somatic symptom disorder - B criteria scale, *VAS*  visual analogue scale,* FEV*_*1*_  forced expiratory volume in 1 s,* VC *  vital capacity, *DLCO * diffusion capacity for carbon monoxide.

Table 4 shows the results of the univariate regression models with DLI as the dependent variable. In PCS patients, SSD (i.e., the combination of SSD-12 and PHQ-15) (Odds ratio 13.8; 95% Confidence Interval 1.7-109.9) and PHQ-9 (10.1; 2.2–47.5) were the most predictive scales for DLI. The self-rated item ´fatigue´ (21.2; 4.1-109.4) was the strongest associated symptom. In asthma, the GAD-7 (15.0; 1.9-116.8) was strongest associated with DLI, followed by FAS and SSD. In patients with COPD, PHQ-9 (8.9; 1.1–71.8) and FAS (6.0; 1.6–21.6) were strongly associated with DLI, and ´shortness of breath´ (10.3; 2,7–39,5) was the strongest associated symptom. Among the lung function parameters, diffusion capacity was associated with DLI in asthma and COPD. In COPD, this also tends to apply to FEV1 (% of predicted) and VC (% of predicted). The interaction analysis did not reveal any significant group differences in the questionnaires; in the case of lung function diagnostics, this only applied to the diffusion capacity (*p* = 0.05).

**Table 4:** Results of the univariate regression models to predict daily life impairment (DLI).

	PCS		Asthma		COPD		Interaction Gruppe x Symptom
Questionnaire	OR (95% CI)	p-value	OR (95% CI)	p-value	OR (95% CI)	p-value	p-value
SSD-12 + PHQ-15	13.8 (1.7-109.9)	0.013	8.5 (2.4–30.1)	< 0.001	1.9 (0.5–7.5)	0.365	0.176
FAS	4.8 (1.4–16.3)	0.012	8.6 (3.6–20.3)	< 0.001	6.0 (1.6–21.6)	0.006	0.832
PHQ-9	10.1 (2.2–47.5)	0.003	5.5 (1.5–19.8)	0.009	8.9 (1.1–71.8)	0.041	0.823
GAD-7	2.8 (0.6–13.3)	0.186	15.0 (1.9-116.8)	0.010	4.2 (0.5–34.7)	0.180	0.440
Symptom							
Fatigue	21.2 (4.1-109.4)	< 0.001	7.0 (3.0-16.4)	< 0.001	2.5 (0.8–8.2)	0.136	0.170
Shortness of breath	12.1 (3.3–44.0)	< 0.001	5.7 (2.4–13.2)	< 0.001	10.3 (2.7–39.5)	< 0.001	0.559
Impaired cognition	14.5 (3.6–57.8)	< 0.001	4.6 (2.0-10.6)	< 0.001	6.1 (1.3–29.7)	0.024	0.382
Lung function							
FEV1%pred.	1.002 (0.975–1.029)	0.906	0.993 (0.975–1.012)	0.453	0.970 (0.941–1.001)	0.056	0.297
VC %pred.	1.002 (0.972–1.034)	0.891	0.984 (0.962–1.006)	0.155	0.961 (0.923–1.001)	0.056	0.273
DLCO %pred.	0.999 (0.971–1.028)	0.946	0.967 (0.943–0.993)	0.012	0.947 (0.916–0.979)	0.001	0.050

*%pred*  percentage of the predicted value,* DLCO*  Diffusion capacity for carbon monoxide,* FAS*  fatigue assessment scale,* FEV1*  forced expiratory volume in 1 s,* GAD-7 *  generalized anxiety disorder 7,* OR*  odds ration,* PCS *  Post-COVID syndrome,* PHQ-9 * Patient health questionnaire 9,* PHQ-15*  Patient health questionnaire 15,*SSD-12 * Somatic symptom disorder - B criteria scale,* VC*  vital capacity.

**Fig. 1:**
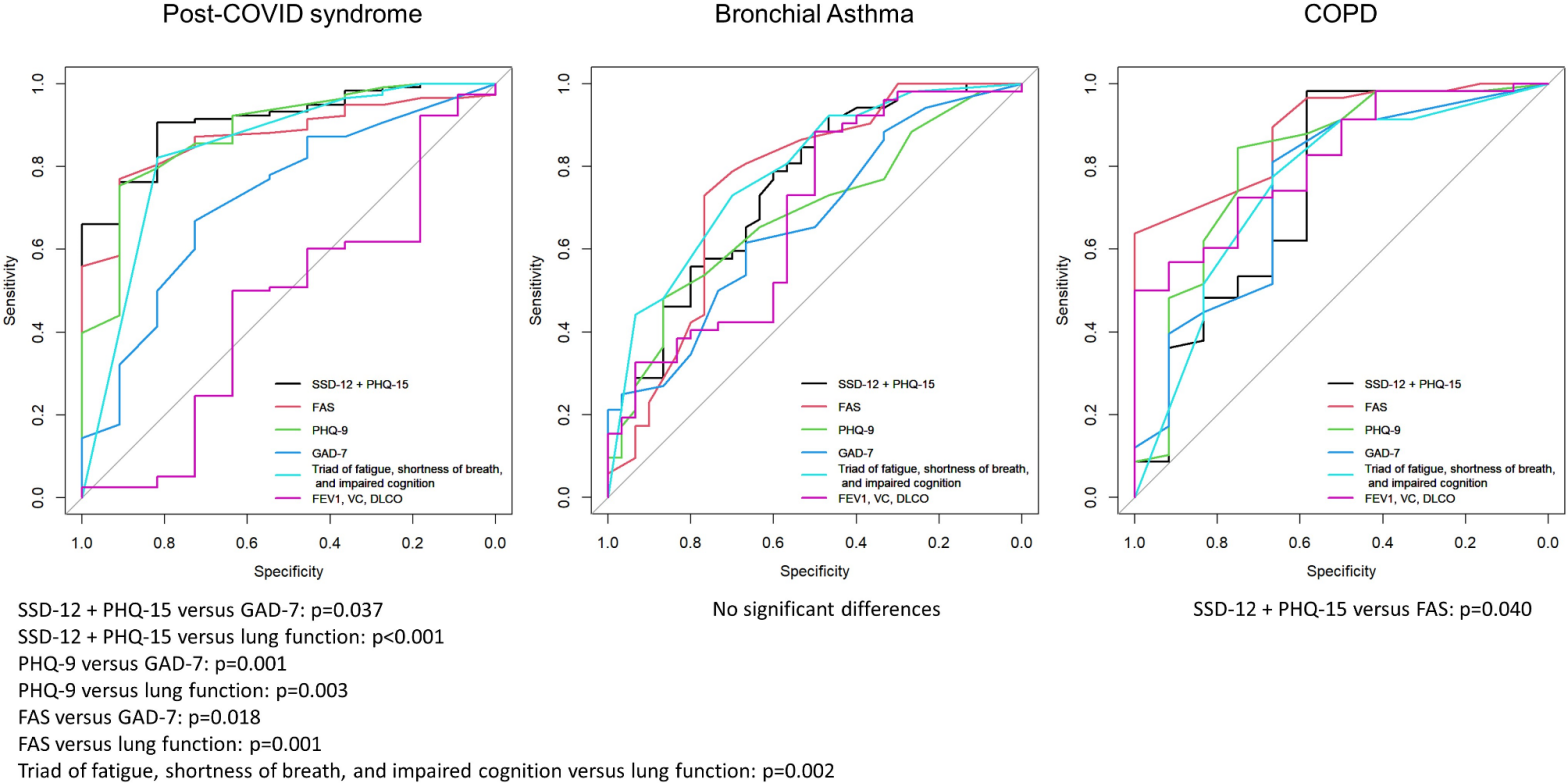
Diagnostic performance of questionnaires, symptoms and lung function parameters for daily life impairment in the PCS, Asthma and COPD groups. The ROC plots sensitivity against specificity using all possible cut-off values of the predictors. The diagnostic accuracy is measured by the areas under the curves (AUC) as presented in Table 4. Curves that extend into the top left corner indicate higher diagnostic performance.* DLCO*  diffusion capacity for carbon monoxide,* FAS*  Fatigue assessment scale,* FEV1*  forced expiratory volume in 1 s,* GAD-7*  generalized anxiety disorder 7,* PHQ-9 * Patient health questionnaire 9,* PHQ-15 * patient health questionnaire 15,* SSD-12 * somatic symptom disorder - B criteria scale,* VC*  vital capacity.

The ROC analysis showed an excellent value of the combination of SSD-12 and PHQ-15 scales for the prediction of DLI in PCS (AUC = 0.991; 95%CI 0.841–0.982), with FAS, PHQ-9, and the triad fatigue, impaired cognition, and dyspnea also showing very good results (Table 5). In patients with asthma, no AUC > 0.80 could be identified. The FAS showed a high value in COPD patients with an AUC = 0.888 (0.798–0.978), as did lung function with an AUC = 0.818 (0.697–0.938). However, the group difference between the patient samples was only significant for lung function (Triad of FEV_1_, VC, DLCO; *p* = 0.003). Significant differences in AUCs within the groups are shown in Fig. 1. All details are available in the online supplement.

**Table 5:** AUC of the ROC analyses for the prediction of daily life impairment in post-COVID syndrome (PCS), asthma, and COPD.

Questionnaire/	PCS (*n* = 129)*	Asthma (*n* = 82)*	COPD (*n* = 70)*
Lung function	AUC (95%CI)	AUC (95%CI)	AUC (95%CI)
SSD-12 + PHQ-15	0.911 (0.841–0.982)	0.748 (0.634–0.862)	0.749 (0.564–0.935)
FAS	0.872 (0.791–0.953)	0.757 (0.638–0.876)	0.888 (0.798–0.978)
PHQ-9	0.811 (0.776–0.986)	0.658 (0.569–0.802)	0.815 (0.655–0.975)
GAD-7	0.722 (0.561–0.884)	0.666 (0.546–0.786)	0.746 (0.576–0.916)
Triad of fatigue, shortness of breath, and impaired cognition	0.840 (0.706–0.973)	0.788 (0.688–0.889)	0.754 (0.588–0.920)
Triad of FEV1, VC, DLCO	0.463 (0.257–0.669)	0.683 (0.558–0.808)	0.818 (0.697–0.938)**

* Complete case analysis of patients with data available for all of the presented variables.

** AUC-difference COPD vs. PCS: -0.355 (95% CI -0.124; -1.845; *p* = 0.003).

*95%CI*  95% confidence interval,* AUC * area under the curve, *DLCO*  diffusion capacity for carbon monoxide,* FAS *  fatigue assessment scale,* FEV1*  forced expiratory volume in 1 s,* GAD-7 * generalized anxiety disorder 7,* PCS * Post-COVID syndrome, *PHQ-9  * patient health questionnaire 9, *PHQ-15*  patient health questionnaire 15, *ROC*  receiver operator characteristic, *SSD-12*  somatic symptom disorder - B criteria scale,* VC* vital capacity.

## Discussion

The comparison between the patient groups showed that PCS patients suffer considerably more frequently from DLI than patients with asthma or COPD. The quality of life in PCS patients was significantly lower, and increased depression and anxiety were more prevalent. In patients with PCS, DLI was most strongly associated with fatigue, impaired cognition, dyspnea, and the SSD-12 and PHQ-15 scales, while in asthma the association with DLI was strongest with the GAD-7. In patients with COPD, DLI was most strongly associated with the PHQ-9 and shortness of breath. In asthma and COPD, but not in PCS, the reduction in diffusion capacity also predicted DLI.

The high proportion of DLI in patients suffering from PCS can probably best be explained by the patient selection itself, as these are patients who were assigned to the rehabilitation program due to their poor state of health. Their significantly reduced quality of life of 0.64 on average in the EQ-5D-5 L is remarkable. In a validation study of the EQ-5D-5 L with asthma patients, 0.82 was the lowest score in patients with high comorbidity, i.e. with at least two chronic conditions^36^. In a study by Nolan et al., the scores of the EQ-5D-5 L in COPD patients ranged from 0.68 to 0.70^32^. This is comparable to the scores of the COPD patients in our study. These numbers illustrate the poor subjective health status of the PCS patients, who were on average 10 years younger than the COPD patients in our study. The considerably higher scores on the depression, anxiety and fatigue scales compared to asthma and COPD underline the poor mental health of this patient group.

There are several explanations why PCS patients are heavily burdened, such as chronic inflammatory processes or autoimmune reactions^9,10^. However, as our in-depth analysis shows, the impact of SSD seems to be of particular importance. In the univariate regression models SSD was more strongly associated with DLI in PCS than in asthma and COPD. It is conceivable that the still unexplained symptoms in PCS cause an intensive preoccupation with the disease, resulting in a higher rating in the somatization scales. Accordingly, the ROC analysis shows only for the PCS group that the association of DLI with SSD is considerably higher than the association with the triad ‘fatigue, shortness of breath, and impaired cognition. In this regard, it must be considered that information is lost when dichotomizing psychometric scales using established cut-off values, whereas the full information content is used in the ROC analysis. Interestingly, lung function parameters and particularly diffusion capacity do not seem to play a role with regards to DLI for PCS patients. This contrasts the results of the study by Kersten et al.^8^ which showed a correlation between well-being and the results of diffusion testing. However, it is important to highlight that the diffusion capacity in our group was higher than in their study, which was conducted at a university post COVID outpatient clinic.

Even though a cross-sectional study is not able to causally explain the identified associations, our data suggest that psychosocial care for patients with PCS should be provided in addition to medical treatment. Beyond that, the data from our study confirm previous findings from the field of psycho-pneumology, which point to characteristic comorbidities in asthma^19,20^ and COPD^22–24^. Therefore, specific psychotherapeutic interventions should be developed depending on the specific disease. Due to its interdisciplinary and multi-professional approach, inpatient pulmonary rehabilitation offers a suitable framework in which holistic treatment for patients with chronic diseases can be implemented^3,37^. In this context, the results can contribute to the further development of treatment options and the creation of disease-specific treatment guidelines, so that the more prevalent psychological distress (e.g. a stronger focus on anxiety disorders in patients with asthma or on SSD in patients with PCS) can be taken in to account more adequately.

### Limitations

An important limitation is the relatively low participation rate, even after a long recruitment period. It is likely that patients with a higher level of suffering took part. However, this would apply to all three groups to a similar extent; and we only used statistics that provide estimates of group contrasts that are robust to the prevalence of the outcome being analyzed. It should be noted that the non-responders in the asthma patients were slightly older on average. This difference might be caused merely by chance. Even though an impact on the study results seems questionable, it is advisable to take the difference into account when interpreting or generalizing the data. Although the regression models and ROC analyses showed clear differences, these were not significant in the interaction analyses, which is most likely due to insufficient statistical power despite the relatively high total number of patients. Even if a clear group specificity cannot be conclusively clarified, the patterns appear plausible and clinically consistent. Due to the small number of PCS patients without DLI, no multivariable models could be calculated, so simultaneous effect estimation and correction for potential confounding variables could not be performed. Another important limitation is the cross-sectional design, which does not allow any causal conclusions or statements about the longitudinal course of the disease. Beyond that, it should be noted that we used questionnaires to diagnose psychological comorbidity. Ideally, the diagnosis would have to be validated by a structured clinical interview. This was not possible due to financial and organizational reasons. However, this should not affect the results of the metric correlations, especially in the ROC analyses. Finally, we could not control for other unknown psychological and socio-cultural variables. Future studies should ideally assess the importance of mental (co-)morbidity during the course of the disease in a longitudinal setting. Furthermore, qualitative studies could help to identify deeper relationships between clinical symptoms, SSD, and other aspects of mental health in patients with PCS.

## Conclusion

The PCS patients at the rehabilitation clinic showed a lower quality of life than patients with asthma or COPD, accompanied by poor mental health. The results suggest that SSD has a high predictive value for DLI in PCS patients. In the context of pulmonary rehabilitation, this should be appropriately taken into account in therapy, for example through concomitant psychotherapy and dosed exercise training. However, the increased psychological comorbidity in the other investigated lung diseases should also be considered and adequately treated in the context of pulmonary rehabilitation. According to our data, this concerns particularly the anxiety component in patients with asthma and depressive disorders in patients with COPD.

## Electronic supplementary material

Below is the link to the electronic supplementary material.

Supplementary Material 1

Supplementary Material 2

## Data Availability

The datasets generated and analysed during the current study are not publicly available due to very strict data protection regulations but are available from the corresponding author on reasonable request.
