# Supplementary material for: The implications of somatic symptom disorder on the impairment of daily life are greater in post-COVID syndrome than in asthma or COPD - results of a cross-sectional study in a rehabilitation clinic
Source: Sci Rep. 2025 Apr 5;15:11719. doi: 10.1038/s41598-025-96055-x (PMC11972386; doi:10.1038/s41598-025-96055-x)
Supplement: Supplementary file 1 — Supplementary Material 1 [file 41598_2025_96055_MOESM1_ESM.html]

Supplementary material to the Scientific Reports article: Lower quality of life and poorer mental health in post-COVID syndrome than in Asthma or COPD - a cross-sectional study 


Code 

- Show All Code
- Hide All Code

# Supplementary material to the Scientific Reports article: Lower quality of life and poorer mental health in post-COVID syndrome than in Asthma or COPD - a cross-sectional study

#### Antonius Schneider, Alexander Hapfelmeier, Anna Greißel, Matthias Limbach, Gabriele Schwarzl, Franziska Ebert, Veronika Huber, Markus C. Hayden

# 1 AUCs (95% CI)

## 1.1 All groups


|  | AUC | Lower | Upper |
| --- | --- | --- | --- |
| SSD-12 + PHQ-15 | 0.820 | 0.755 | 0.884 |
| FAS | 0.837 | 0.778 | 0.896 |
| PHQ-9 | 0.813 | 0.752 | 0.874 |
| GAD-7 | 0.709 | 0.633 | 0.785 |
| Triad of fatigue, shortness of breath, and impaired cognition | 0.825 | 0.763 | 0.887 |
| FEV1, VC, DLCO | 0.702 | 0.622 | 0.781 |

## 1.2 PCS

|  | AUC | Lower | Upper |
| --- | --- | --- | --- |
| SSD-12 + PHQ-15 | 0.911 | 0.841 | 0.982 |
| FAS | 0.872 | 0.791 | 0.953 |
| PHQ-9 | 0.881 | 0.776 | 0.986 |
| GAD-7 | 0.722 | 0.561 | 0.884 |
| Triad of fatigue, shortness of breath, and impaired cognition | 0.840 | 0.706 | 0.973 |
| FEV1, VC, DLCO | 0.463 | 0.257 | 0.669 |

## 1.3 Asthma

|  | AUC | Lower | Upper |
| --- | --- | --- | --- |
| SSD-12 + PHQ-15 | 0.748 | 0.634 | 0.862 |
| FAS | 0.757 | 0.638 | 0.876 |
| PHQ-9 | 0.685 | 0.569 | 0.802 |
| GAD-7 | 0.666 | 0.546 | 0.786 |
| Triad of fatigue, shortness of breath, and impaired cognition | 0.788 | 0.688 | 0.889 |
| FEV1, VC, DLCO | 0.683 | 0.558 | 0.808 |

## 1.4 COPD

|  | AUC | Lower | Upper |
| --- | --- | --- | --- |
| SSD-12 + PHQ-15 | 0.749 | 0.564 | 0.935 |
| FAS | 0.888 | 0.798 | 0.978 |
| PHQ-9 | 0.815 | 0.655 | 0.975 |
| GAD-7 | 0.746 | 0.576 | 0.916 |
| Triad of fatigue, shortness of breath, and impaired cognition | 0.754 | 0.588 | 0.920 |
| FEV1, VC, DLCO | 0.818 | 0.697 | 0.938 |

# 2 ROC Plots

## 2.1 All groups

## 2.2 PCS

## 2.3 Asthma

## 2.4 COPD

# 3 Tests

## 3.1 Comparison of groups by predictor

### 3.1.1 PCS vs. Asthma


|  | AUC of PCS | AUC of Asthma | Diff | Lower | Upper | pval |
| --- | --- | --- | --- | --- | --- | --- |
| SSD-12 + PHQ-15 | 0.911 | 0.748 | 0.164 | 0.029 | 0.299 | 0.018 |
| FAS | 0.872 | 0.757 | 0.115 | -0.031 | 0.260 | 0.121 |
| PHQ-9 | 0.881 | 0.685 | 0.196 | 0.037 | 0.354 | 0.016 |
| GAD-7 | 0.722 | 0.666 | 0.056 | -0.146 | 0.259 | 0.585 |
| Triad of fatigue, shortness of breath, and impaired cognition | 0.840 | 0.788 | 0.052 | -0.116 | 0.219 | 0.545 |
| FEV1, VC, DLCO | 0.463 | 0.683 | -0.220 | 0.012 | -1.727 | 0.063 |

### 3.1.2 PCS vs. COPD


|  | AUC of PCS | AUC of COPD | Diff | Lower | Upper | pval |
| --- | --- | --- | --- | --- | --- | --- |
| SSD-12 + PHQ-15 | 0.911 | 0.749 | 0.162 | -0.039 | 0.363 | 0.113 |
| FAS | 0.872 | 0.888 | -0.016 | 0.106 | -0.138 | 0.793 |
| PHQ-9 | 0.881 | 0.815 | 0.066 | -0.127 | 0.260 | 0.499 |
| GAD-7 | 0.722 | 0.746 | -0.024 | 0.212 | -0.260 | 0.840 |
| Triad of fatigue, shortness of breath, and impaired cognition | 0.840 | 0.754 | 0.085 | -0.129 | 0.300 | 0.432 |
| FEV1, VC, DLCO | 0.463 | 0.818 | -0.355 | -0.124 | -1.847 | 0.003 |

### 3.1.3 Asthma vs. COPD


|  | AUC of Asthma | AUC of COPD | Diff | Lower | Upper | pval |
| --- | --- | --- | --- | --- | --- | --- |
| SSD-12 + PHQ-15 | 0.748 | 0.749 | -0.002 | 0.218 | -0.221 | 0.989 |
| FAS | 0.757 | 0.888 | -0.131 | 0.020 | -0.282 | 0.088 |
| PHQ-9 | 0.685 | 0.815 | -0.129 | 0.070 | -0.329 | 0.202 |
| GAD-7 | 0.666 | 0.746 | -0.080 | 0.130 | -0.291 | 0.451 |
| Triad of fatigue, shortness of breath, and impaired cognition | 0.788 | 0.754 | 0.034 | -0.162 | 0.230 | 0.733 |
| FEV1, VC, DLCO | 0.683 | 0.818 | -0.134 | 0.040 | -0.309 | 0.131 |

## 3.2 Comparison of predictors by group

### 3.2.1 PCS


|  | AUC of roc1 | AUC of roc2 | Diff | Lower | Upper | pval |
| --- | --- | --- | --- | --- | --- | --- |
| SSD-12 + PHQ-15 vs. FAS | 0.911 | 0.872 | 0.040 | -0.049 | 0.128 | 0.379 |
| SSD-12 + PHQ-15 vs. PHQ-9 | 0.911 | 0.881 | 0.030 | -0.088 | 0.149 | 0.615 |
| SSD-12 + PHQ-15 vs. GAD-7 | 0.911 | 0.722 | 0.189 | 0.011 | 0.367 | 0.037 |
| SSD-12 + PHQ-15 vs. Triad of fatigue, shortness of breath, and impaired cognition | 0.911 | 0.840 | 0.072 | -0.033 | 0.176 | 0.178 |
| SSD-12 + PHQ-15 vs. FEV1, VC, DLCO | 0.911 | 0.463 | 0.448 | 0.245 | 0.652 | 0.000 |
| FAS vs. PHQ-9 | 0.872 | 0.881 | -0.009 | 0.047 | -0.065 | 0.747 |
| FAS vs. GAD-7 | 0.872 | 0.722 | 0.149 | 0.025 | 0.273 | 0.018 |
| FAS vs. Triad of fatigue, shortness of breath, and impaired cognition | 0.872 | 0.840 | 0.032 | -0.108 | 0.172 | 0.656 |
| FAS vs. FEV1, VC, DLCO | 0.872 | 0.463 | 0.409 | 0.156 | 0.661 | 0.002 |
| PHQ-9 vs. GAD-7 | 0.881 | 0.722 | 0.159 | 0.063 | 0.255 | 0.001 |
| PHQ-9 vs. Triad of fatigue, shortness of breath, and impaired cognition | 0.881 | 0.840 | 0.041 | -0.114 | 0.196 | 0.602 |
| PHQ-9 vs. FEV1, VC, DLCO | 0.881 | 0.463 | 0.418 | 0.150 | 0.686 | 0.002 |
| GAD-7 vs. Triad of fatigue, shortness of breath, and impaired cognition | 0.722 | 0.840 | -0.117 | 0.088 | -0.323 | 0.263 |
| GAD-7 vs. FEV1, VC, DLCO | 0.722 | 0.463 | 0.259 | -0.071 | 0.589 | 0.123 |
| Triad of fatigue, shortness of breath, and impaired cognition vs. FEV1, VC, DLCO | 0.840 | 0.463 | 0.377 | 0.130 | 0.623 | 0.003 |

### 3.2.2 Asthma


|  | AUC of roc1 | AUC of roc2 | Diff | Lower | Upper | pval |
| --- | --- | --- | --- | --- | --- | --- |
| SSD-12 + PHQ-15 vs. FAS | 0.748 | 0.757 | -0.009 | 0.084 | -0.102 | 0.845 |
| SSD-12 + PHQ-15 vs. PHQ-9 | 0.748 | 0.685 | 0.062 | -0.025 | 0.150 | 0.160 |
| SSD-12 + PHQ-15 vs. GAD-7 | 0.748 | 0.666 | 0.082 | -0.010 | 0.174 | 0.082 |
| SSD-12 + PHQ-15 vs. Triad of fatigue, shortness of breath, and impaired cognition | 0.748 | 0.788 | -0.040 | 0.078 | -0.159 | 0.504 |
| SSD-12 + PHQ-15 vs. FEV1, VC, DLCO | 0.748 | 0.683 | 0.064 | -0.063 | 0.192 | 0.322 |
| FAS vs. PHQ-9 | 0.757 | 0.685 | 0.072 | -0.022 | 0.166 | 0.136 |
| FAS vs. GAD-7 | 0.757 | 0.666 | 0.091 | -0.008 | 0.190 | 0.071 |
| FAS vs. Triad of fatigue, shortness of breath, and impaired cognition | 0.757 | 0.788 | -0.031 | 0.117 | -0.179 | 0.681 |
| FAS vs. FEV1, VC, DLCO | 0.757 | 0.683 | 0.074 | -0.062 | 0.210 | 0.288 |
| PHQ-9 vs. GAD-7 | 0.685 | 0.666 | 0.019 | -0.065 | 0.104 | 0.656 |
| PHQ-9 vs. Triad of fatigue, shortness of breath, and impaired cognition | 0.685 | 0.788 | -0.103 | 0.029 | -0.235 | 0.127 |
| PHQ-9 vs. FEV1, VC, DLCO | 0.685 | 0.683 | 0.002 | -0.135 | 0.139 | 0.978 |
| GAD-7 vs. Triad of fatigue, shortness of breath, and impaired cognition | 0.666 | 0.788 | -0.122 | 0.006 | -0.250 | 0.061 |
| GAD-7 vs. FEV1, VC, DLCO | 0.666 | 0.683 | -0.017 | 0.122 | -0.157 | 0.808 |
| Triad of fatigue, shortness of breath, and impaired cognition vs. FEV1, VC, DLCO | 0.788 | 0.683 | 0.105 | -0.056 | 0.265 | 0.200 |

### 3.2.3 COPD


|  | AUC of roc1 | AUC of roc2 | Diff | Lower | Upper | pval |
| --- | --- | --- | --- | --- | --- | --- |
| SSD-12 + PHQ-15 vs. FAS | 0.749 | 0.888 | -0.139 | -0.007 | -0.271 | 0.040 |
| SSD-12 + PHQ-15 vs. PHQ-9 | 0.749 | 0.815 | -0.065 | 0.040 | -0.171 | 0.224 |
| SSD-12 + PHQ-15 vs. GAD-7 | 0.749 | 0.746 | 0.003 | -0.127 | 0.133 | 0.965 |
| SSD-12 + PHQ-15 vs. Triad of fatigue, shortness of breath, and impaired cognition | 0.749 | 0.754 | -0.005 | 0.175 | -0.185 | 0.956 |
| SSD-12 + PHQ-15 vs. FEV1, VC, DLCO | 0.749 | 0.818 | -0.068 | 0.132 | -0.269 | 0.505 |
| FAS vs. PHQ-9 | 0.888 | 0.815 | 0.073 | -0.058 | 0.205 | 0.276 |
| FAS vs. GAD-7 | 0.888 | 0.746 | 0.142 | -0.006 | 0.289 | 0.060 |
| FAS vs. Triad of fatigue, shortness of breath, and impaired cognition | 0.888 | 0.754 | 0.134 | -0.021 | 0.288 | 0.090 |
| FAS vs. FEV1, VC, DLCO | 0.888 | 0.818 | 0.070 | -0.083 | 0.224 | 0.368 |
| PHQ-9 vs. GAD-7 | 0.815 | 0.746 | 0.068 | -0.047 | 0.184 | 0.247 |
| PHQ-9 vs. Triad of fatigue, shortness of breath, and impaired cognition | 0.815 | 0.754 | 0.060 | -0.105 | 0.226 | 0.475 |
| PHQ-9 vs. FEV1, VC, DLCO | 0.815 | 0.818 | -0.003 | 0.188 | -0.194 | 0.976 |
| GAD-7 vs. Triad of fatigue, shortness of breath, and impaired cognition | 0.746 | 0.754 | -0.008 | 0.203 | -0.219 | 0.942 |
| GAD-7 vs. FEV1, VC, DLCO | 0.746 | 0.818 | -0.071 | 0.123 | -0.265 | 0.472 |
| Triad of fatigue, shortness of breath, and impaired cognition vs. FEV1, VC, DLCO | 0.754 | 0.818 | -0.063 | 0.101 | -0.227 | 0.450 |
